# Hospital Meetings, &C.

**Published:** 1898-04-23

**Authors:** 


### HOSPITAL MEETINGS, &C.

### METROPOLITAN HOSPITAL.

The Lord Mayor, who is *ex officio* the president of the institution, occupied the chair at the annual festival dinner of the Metropolitan Hospital, which took place on Monday, the 18th inst., at the Whitehall Rooms. He was supported by Sheriffs Green and Dewar, Mr. C. J. Thomas (the chairman of the hospital), Mr. Ernest Flower, M.P., Mr. John Lowles, M.P., and about 120 other gentlemen interested in the hospital. The Lord Mayor, in proposing the toast of the evening, "Prosperity to the Hospital," said he associated himself the more closely with the hospital as it was first established in the ward over which he had the honour to preside. The hospital was languishing for want of funds, it owed its tradesmen something like £6,700 (an increase of £1,700 on the previous year), it was burdened with a mortgage to the tune of £10,000, and it had an overdraft at the bank of about £4,000. Besides this, it had to provide for a constantly increasing number of patients, and he therefore appealed most earnestly to the charitable public for the funds to wipe off its debts, and to enable it more fully to undertake the work which was so necessary in the poor and crowded neighbourhood in which the hospital was situated. Mr. C. J. Thomas, in responding, referred to the provident system in vogue at the hospital, under which the working men of the neighbourhood subscribed a small sum weekly during health in order to provide for their relief in the time of sickness. Out of the 100,000 attendances made by the out-patients, about 28,000 attendances were made under this system. He did not believe that so far as organisation, nursing, and medical attendance were concerned, anything would be found wanting at the Metropolitan Hospital, which was situated in the position where it was most needed, but in the position where it was overlooked by wealthy, charitable people, and therefore in the position where it was most difficult to obtain the funds to keep it in working order. The health of the committee of management having been proposed by Alderman and Sheriff Green, and responded to by Lieut.-Colonel Montefiore, the Secretary read the list of the contributions received as a result of the dinner. They amounted to £2,139, in which was included a sum of £50 a-year to be given during 1898, 1899, and 1900, on condition that it was used towards paying the premiums of the hospital's nurses in the Royal National Pension Fund. Mr. Ernest Flower, M.P., in proposing "The Hospital Staff," paid a tribute to the splendid effort made by the Prince of Wales to clear the metropolitan Hospitals of debt. Dr. A. T. Davies and Mr. D. H. Goodsall responded. Other toasts followed.

